# Preoperative Misdiagnosis in Patients Undergoing Pancreatoduodenectomy

**DOI:** 10.3390/jcm11237200

**Published:** 2022-12-03

**Authors:** Elena Panettieri, Alessandro Coppola

**Affiliations:** 1 Hepatobiliary Surgery Unit, Fondazione Policlinico A. Gemelli IRCCS, Università del Sacro Cuore, 00168 Rome, Italy; 2 Dipartimento di Chirurgia, Sapienza Università di Roma, 00185 Rome, Italy

Distal bile duct cholangiocarcinoma, ampullary adenocarcinoma, duodenal carcinoma, and ductal adenocarcinoma (PDAC) of the head of the pancreas usually have similar clinical presentation since they arise in the same circumscribed anatomical area, within 2 cm of the major duodenal papilla. For this reason, it is not uncommon to experience misdiagnosis during the diagnostic workup [1,2].

Looking at the estimated rate of new cancer cases per year, PDAC is the periampullary neoplasm with the highest frequency. In fact, it is estimated that PDAC represents 3% of all malignant neoplasms, both in men, where it ranks tenth, and in women, where it represents the eighth most frequent cancer.

Analyzing the data regarding annual mortality, PDAC jumps to fourth place in both sexes, constituting approximately 8% of total deaths from cancer. However, cholangiocarcinoma, despite its low incidence, is also one of the most lethal neoplasms. In males, it ranks fifth, constituting 6% of all cancer deaths, while it ranks seventh in females, at 4% [3].

Therefore, given their relatively low incidence, correct diagnosis is fundamental given the high mortality rate caused by these neoplasms. The only way to improve survival is to ensure correct diagnosis in order to subject patients to the most appropriate therapeutic pathways.

Although these malignancies are all resectable through pancreatoduodenectomy, their prognosis and therapeutic options are different. Given the increasingly relevant role of neoadjuvant chemotherapy in the multimodal treatment of these patients, it is crucial to avoid misdiagnosis, in order to provide patients with the optimal tailored therapeutic approach.

In particular, preoperative chemotherapy (with or without concomitant radiation) is accepted for patients with both resectable and borderline resectable PDAC, with the aim of (i) downsizing the tumor, (ii) increasing the chances of obtaining a radical resection, (iii) selecting patients with a more favorable tumor biology, and (iv) balancing the role of adjuvant chemotherapy [4,5]. Few reports describe the increased use of chemoradiation for nonpancreatic periampullary tumors and its impact on survival [6,7]. Nevertheless, it is important to note not only that the administration of preoperative chemotherapy is less established in this setting, but also that the available regimens are different to those utilized for PDAC. More specifically, fluorouracil, capecitabine, and gemcitabine are the most common agents administered in patients affected by nonpancreatic periampullary adenocarcinoma. On the other hand, oxaliplatin, irinotecan, 5-fluorouracil, and leucovorin (FOLFIRINOX), or gemcitabine with albumin-bound paclitaxel, are the options available for PDAC.

Based on this information, the importance of avoiding preoperative misdiagnosis during the clinical–therapeutic workup of pancreatoduodenectomy candidates is clear.

Abdominal ultrasound is usually the first imaging test performed in patients with painless jaundice. Contrast-enhanced computed tomography (CT) is the most popular imaging tool for use as a second step due to its widespread availability and most radiologists’ familiarity with the technique. Magnetic resonance imaging (MRI) with cholangiopancreatography (MRCP) can be used where access and expertise is available. [8]. Notably, MRI offers better soft-tissue characterization, and can more efficiently detect small or isoattenuating masses, which are not easily detected using CT. [9] For this reason, the staging of PDAC has become the diagnostic modality of choice at some institutions [10]. Other conditions, both benign and malignant, may mimic PDAC, especially when small in size, since adenocarcinomas of the ampullary region show similar signal characteristics and enhancement properties [10]. The association of MRI with MRCP allows for a thorough evaluation of the biliary tree and the ampullary area, avoiding the post-procedural risks associated with endoscopic retrograde cholangiopancreatography (ERCP). Nevertheless, it is important to note that CT offers the most accurate measurement of PDAC size during the pancreatic parenchymal phase, while MRI results in significant underestimation of PDAC size [11].

As for other hepatobiliary diseases [12], it is evident that to maximize the level of accuracy of these diagnostic images, they should be reviewed by radiologists specialized in these diseases and discussed in multidisciplinary meetings.

Endoscopic ultrasonography (EUS) is an important tool for the clinical evaluation and differential diagnosis of PDAC, with a median sensitivity for the detection of pancreatic tumors of 94% [13]. In particular, EUS-fine-needle aspiration (EUS-FNA) has a sensitivity and specificity of 85–92% and 96–98%, respectively [14].

The main role of ERCP is to relieve biliary obstruction, but it can also give important diagnostic information. The fluoroscopic aspect of the biliary stricture can help in the differential diagnostic process. ERCP-guided brushing and biopsy have high specificity for diagnosing malignancy (approaching 95%), with very low sensitivity (23–56% for biliary brushing and 33–65% for fluoroscopic biopsy) [14], with EUS-FNAB being superior. [15,16]

Unfortunately, neoplastic markers such as Ca 19.9 and CEA cannot be considered reliable aids to discriminate between PDAC and other periampullary tumors. The role of Ca 19.9 in PDAC has been, and still is, widely debated in the literature [17]. Its role is important with regard to preoperative staging, possible indication, and the response to neoadjuvant treatments. In addition, it may be useful for predicting outcomes such as lymph node positivity [18], margins, and the risk of disease recurrence. Unfortunately it does not play the same role in the management of other periampullary cancers. The presence of elevated values of Ca 19.9 makes the diagnosis of PDAC more likely than other cancers. However, low levels of Ca 19.9 cannot exclude the diagnosis of PDAC, as a high percentage of patients have low values of the marker and, moreover, about 10% do not express the marker at all.

CEA, alone or in association with other markers, is also unable to help in the differential diagnosis of these tumors [19].

To our knowledge, only one study has specifically focused on the rate and impact of the preoperative misdiagnosis of PDAC and periampullary cancer in patients undergoing pancreatoduodenectomy [1]. Despite the advances in diagnostic resources, the authors showed that 16% of 1244 patients undergoing pancreatoduodenectomy had a clinically relevant preoperative misdiagnosis. Among 679 patients with a final diagnosis of PDAC, 6% were preoperatively misdiagnosed with cholangiocarcinoma, 4% with ampullary cancer, and 2.4% with duodenal cancer. After multivariable analysis, factors predictive of PDAC, compared to periampullary cancer, were weight loss > 10%, a CA 19.9 level > 160 u/mL, any vascular involvement on imaging, a tumor size > 20 mm, and positive pathology obtained using endoscopic ultrasound fine-needle aspiration. Patients with a missed preoperative diagnosis of PDAC had a similar median overall survival (OS) to patients correctly diagnosed with PDAC who did not receive neoadjuvant therapy (21.5 vs. 19.4 months, *p* = 0.08). Patients with PDAC who received neoadjuvant therapy had a significantly longer median OS (25.9 months) than patients with correctly diagnosed PDAC who did not receive preoperative therapy (*p* = 0.021); however, their median OS was not different to that of patients with missed PDAC (*p* = 0.69). Nevertheless, the authors did not focus on the accuracy of preoperative staging and the diagnostic discrepancy between diagnostic modalities (especially radiologic vs. interventional).

Given the importance of understanding which strategies will minimize these diagnostic errors, future studies should consider analyzing the impacts of these misdiagnoses on patient outcomes in larger cohorts of patients. 
